# Supplementary material for: Strategies for motion- and respiration-robust estimation of fMRI intrinsic neural timescales
Source: Imaging Neurosci (Camb). 2024 Oct 28;2:imag-2-00326. doi: 10.1162/imag_a_00326 (PMC12094611; doi:10.1162/imag_a_00326)
Supplement: Supplementary Material [file imag_a_00326-supp.pdf]

SUPPLEMENT

Strategies for motion- and respiration-robust estimation of fMRI intrinsic neural timescales

Andrew Goldberg[1], Isabella Rosario[1], Jonathan Power[2], Guillermo Horga[1,3*], and Kenneth Wengler[1,3,4,5*]

1. New York State Psychiatric Institute, New York, NY, USA
2. Department of Psychiatry, Weill Cornell Medicine, New York, NY USA
3. Department of Psychiatry, Columbia University, New York, NY USA
4. Department of Psychiatry, Icahn School of Medicine at Mount Sinai, New York, NY USA
5. Department of Diagnostic, Molecular and Interventional Radiology, Icahn School of Medicine at Mount Sinai, New York, NY USA

*These authors contributed equally

Analyses comparing Lomb-Scargle interpolation and linear interpolation

Given that linear interpolation is commonly used in post-processing pipelines for resting-state functional magnetic resonance imaging (rs-fMRI) functional connectivity analyses we performed analyses comparing it to Lomb-Scargle (L-S) interpolation. We restricted these analyses to the *L-S interpolation* autocorrelation function (ACF) estimation approach (see Methods and Figure 1 in the main text) where the interpolated data (after high-motion frame censoring) are retained in the ACF estimation. This was done for two reasons: 1) the *L-S interpolation* approach was found to be superior to the *contiguous blocks* approach; and 2) in the *contiguous blocks* approach the interpolated data are only used for bandpass filtering and subsequently excluded from the ACF estimation.

First, we tested the performance of high-motion frame censoring with linear interpolation in removing head motion and respiration artifacts by comparing the similarity—root-mean squared error (RMSE) and Pearson correlation *(r)*—between non-clean runs and their respective clean runs (see Eqs. (5) and (6) in the main text). Compared to the base post-processing, frame censoring with linear interpolation—in contrast to L-S interpolation—did not significantly improve the similarity between non-clean runs and their respective clean run (RMSE: $t_{224} = 0.97$, $P = 0.3336$; Pearson's r: $t_{224} = 1.76$, $P = 0.0795$) but—like L-S interpolation—global signal regression (GSR) and frame censoring together did (RMSE: $t_{224} = -3.08$, $P = 0.0023$; Pearson's r: $t_{224} = 3.81$, $P = 0.0002$).

Next, we compared the similarity between non-clean runs and their respective clean runs when using either L-S or linear interpolation for high-motion frame censoring and either alone or in conjunction with GSR using the same linear mixed-effects (LME) models that were used to assess the performance of GSR and high-motion frame censoring. When GSR was not included (i.e., base post-processing + high-motion frame censoring), for the combined non-clean runs the mean RMSE and r were as follows: L-S interpolation (RMSE = 177.7 $\pm$ 55.8 ms [mean $\pm$ standard deviation]; r = 0.73 $\pm$ 0.12); linear interpolation (RMSE = 224.8 $\pm$ 131.1 ms; r = 0.71 $\pm$ 0.15); with significantly greater dissimilarity for linear vs L-S interpolation (RMSE comparison: t_{224} = 3.40, P = 0.0008; r comparison: t_{224} = -3.50, P = 0.0006). Results when GSR was included (i.e., base post-processing + GSR + high-motion frame censoring): L-S interpolation (RMSE = 163.0 $\pm$ 51.2 ms; r = 0.75 $\pm$ 0.10); linear interpolation (RMSE = 186.2 $\pm$ 108.4 ms; r = 0.74 $\pm$ 0.11); with significantly greater dissimilarity for linear vs L-S interpolation (RMSE comparison: t_{224} = 2.28, P = 0.0235; r comparison: t_{224} = -2.66, P = 0.0083).

Together, these results suggest: 1) that high-motion frame censoring with linear interpolation should not be used without GSR—potentially due to the interpolation of temporally local global signals increasing INT estimation error, whereas L-S interpolation may avoid this problem by using the frequency content of the entire time series; and 2) high-motion frame censoring with linear interpolation when combined with GSR significantly reduces head motion and respiration artifacts but is inferior to L-S interpolation.

Performance of GSR in subtypes of non-clean runs

We performed post-hoc analyses in individual subtypes of non-clean runs given the null finding that GSR on its own (i.e., base post-processing + GSR) did not improve the similarity between non-clean runs and their respective clean run. Table S1 lists the results of these post-hoc analyses. In summary GSR on its own did not improve similarity compared to the base post-processing for any subtype of non-clean runs but when combined with high-motion frame censoring, it was particular effective for deep breaths, both bursts and deep breaths, and high-motion runs.

Table S1. Post-hoc analyses of GSR performance in subtypes of non-clean runs.

	Base vs GSR				+Censoring vs +GSR+Censoring			
	RMSE		r		RMSE		r	
	t^a	P	t^a	P	t^a	P	t^a	P
Burst	-0.79	0.4317	0.01	0.9812	-2.29	0.0255	0.51	0.6114
Deep breaths	1.40	0.1646	-1.56	0.1235	-2.47	0.0158	2.29	0.0251
Both bursts and deep breaths	-2.29	0.0274	1.64	0.1089	-3.78	0.0005	1.82	0.0763
Low motion	-0.92	0.3629	0.62	0.5382	-1.51	0.1408	1.77	0.0859
High motion	-0.29	0.7740	-0.61	0.5462	-3.22	0.0029	1.42	0.1667

[a]The degrees of freedom of the reported t-statistics are as follows: bursts, 58; deep breaths, 70; both bursts and deep breaths, 38; low motion 32; high motion, 32.
GSR, global signal regression; RMSE, root-mean squared error; *r*, Pearson's correlation

Analyses restricted to subcortical parcels

Due to their distinct anatomical locations within the brain, it is possible that respiration and head-motion artifacts could have differential effects on cortical and subcortical brain regions. To investigate this, we performed analyses separately in the 360 cortical parcels (for which results are similar to the whole-brain results; see below) and the 19 subcortical parcels. Within the subcortical parcels, the combined non-clean runs (ΔINT = 25.1 $\pm$ 57.3 ms; t_{157} = 3.11, $P_{\text{Bonferroni}}$ = 0.0130), bursts (ΔINT = 27.8 $\pm$ 35.9 ms; t_{74} = 3.42, $P_{\text{Bonferroni}}$ = 0.0062), both bursts and deep breaths (ΔINT = 38.0 $\pm$ 53.0 ms; t_{64} = 2.75, $P_{\text{Bonferroni}}$ = 0.0468), and high motion (ΔINT = 77.3 $\pm$ 93.9 ms; t_{61} = 3.05, $P_{\text{Bonferroni}}$ = 0.0205) runs showed general increases in average INT across the brain compared to the clean runs. In contrast, the deep breath (ΔINT = 31.5 $\pm$ 75.4 ms; t_{80} = 2.15, $P_{\text{Bonferroni}}$ = 0.2065) and low motion (ΔINT = 0.1 $\pm$ 33.1 ms; t_{61} = 0.14, $P_{\text{Bonferroni}}$ = 1) runs did not show significant differences in average INT compared to the clean runs. While these findings are similar to those reported for the whole-brain analyses (and the cortical-parcels-only analyses; see below) in terms of the direction and significance of effects, the magnitude of effects is nominally smaller in the subcortical parcels.

To better understand the differences between subcortical and cortical parcels in INT estimation error introduced by respiration and head-motion artifacts, we compared RMSE calculated separately for cortical parcels (Eq. (5) in Main Text with p going from 1 to 360) and subcortical parcels (Eq. (5) in Main Text with p going from 361 to 379) for the different types of non-clean runs. Here, we observed lower RMSE in subcortical compared to cortical parcels for all types of non-clean runs compared to their respective clean run (combined non-clean: t_{112} = -20.05; burst: t_{112} = -9.08; deep breaths: t_{112} = -13.01; both bursts and deep breaths: t_{112} = -12.15; low motion: t_{112} = -12.28; high motion: t_{112} = -9.48; paired t-tests; all $P_{\text{Bonferroni}} < 0.0001$

corrected for 6 comparisons). Finally, we compared the ability of high-motion frame censoring and GSR on mitigating the effects of respiration and head-motion artifacts between subcortical and cortical parcels by comparing the relative amount of reduction in RMSE following high-motion frame censoring and GSR (calculated as a percent difference in the RMSE with and without high-motion frame censoring and GSR to account for the overall lower RMSE in subcortical parcels). Here, we observed no significant difference in the relative reduction in RMSE between subcortical and cortical parcels (combined non-clean: t_{112} = -2.55; burst: t_{112} = -1.00; deep breaths: t_{112} = -1.56; both bursts and deep breaths: t_{112} = -1.53; low motion: t_{112} = -1.40; high motion: t_{112} = 0.16; paired t-tests; all $P_{\text{Bonferroni}} > 0.0717$ corrected for 6 comparisons).

Together, these results suggest that, compared to cortical brain regions, subcortical brain regions are generally less sensitive to the effects of respiration and head-motion artifacts, and that high-motion frame censoring and GSR are similarly effective at reducing these effects.

Analyses restricted to cortical parcels

Similar to the whole-brain analysis (and as expected given the cortical parcels comprise 360 out of 379 total parcels; 95%), within the cortical parcels, the combined non-clean runs (ΔINT = 60.1 $\pm$ 129.9 ms; t_{157} = 3.65, $P_{\text{Bonferroni}}$ = 0.0021), bursts (ΔINT = 66.9 $\pm$ 161.8 ms; t_{74} = 3.92, $P_{\text{Bonferroni}}$ = 0.0012), both bursts and deep breaths (ΔINT = 138.2 $\pm$ 120.8 ms; t_{64} = 5.13, $P_{\text{Bonferroni}} <$ 0.0001), and high motion (ΔINT = 121.8 $\pm$ 158.6 ms; t_{61} = 3.30, $P_{\text{Bonferroni}}$ = 0.0097) runs showed general increases in average INT across the brain compared to the clean runs (Figure 3B). In contrast, the deep breath (ΔINT = 44.8 $\pm$ 109.7 ms; t_{80} = 1.84, $P_{\text{Bonferroni}}$ = 0.4135) and low motion (ΔINT = 30.7 $\pm$ 100.4 ms; t_{61} = 1.33, $P_{\text{Bonferroni}}$ = 1) runs did not show significant differences in average INT compared to the clean runs. Non-clean runs with more motion were more dissimilar to the clean run from the same subject (relationship between mean FD and RMSE: t_{111} = 3.16, P = 0.0020; relationship between mean FD and Pearson's r: t_{111} = -1.94, P = 0.0543).
